# Effect of laser irradiated silver doped polystyrene/polyethylene terephthalate (PET) thin film for solar cell applications

**DOI:** 10.1039/d2ra04777b

**Published:** 2022-11-16

**Authors:** Jibrin Alhaji Yabagi, Muhammad Hasnain Jameel, Abdullah Hasan Jabbar, Mohammed Isah Kimpa, Rami Qays Malik, Sim Pei Xin, Ndanusa Babakatcha, Muhammad Bello Ladan, Maytham Qabel Hamzah, MohdArif Agam, M. M. Hessien, Gaber A. M. Mersal

**Affiliations:** Departments of Physics, Faculty of Natural Sciences, Ibrahim Badamasi Babangida University Lapai P.M.B 11, Lapai Niger State Nigeria jibrinyabagi@ibbu.edu.ng; Department of Physics and Chemistry, University Tun Hussein Onn Malaysia 84600 Pagoh Muar Johor Malaysia ​arif@uthm.edu.my; Optical Department, College of Medical and Health Technology, Sawa University, Ministry of Higher Education and Scientific Research Samawah Al-Muthanaa Iraq; Department of Physics, School of Physical Sciences, Federal University of Technology Minna P.M.B. 65 Minna Niger State Nigeria; Medical Instrumentation Techniques Engineering Department, Al-Mustaqbal University College Babylon Iraq; Directorate of Education Al-Muthanna, Ministry of Education Republic of Iraq; Department of Chemistry, College of Science, Taif University P.O Box 11099 Taif 21944 Saudi Arabia

## Abstract

In the current research, the resist action of silver-doped polystyrene/polyethylene terephthalate (PET) solar thin film towards laser irradiation was observed. Moreover, silver-doped polystyrene nanoparticles were synthesized *via* a chemical technique while the PET film was purchased from the commercial market. Nd:YAG pulsed laser has been used to irradiate the samples at 2 minutes, 4 minutes, and 6 minutes respectively. The XRD (X-ray diffraction) pattern shows that silver-doped polystyrene peak at around angle *θ* = 26° tends to decrease after the bombardment of Nd:YAG pulsed laser. This indicates that the crystallinity of PET film decreased after laser irradiation. The Raman spectra have revealed the zwitter characteristics of silver-doped polystyrene are shifting of bands at 1380 cm^−1^ and 1560 cm^−1^ upon laser irradiation. For PET film, the Raman spectra showed that the exposed regions tend to change to cross-linking/chain-scissoring at 2 minutes and 4 minutes of irradiation. The surface roughness first increases and decreases upon irradiation. These results indicate that silver-doped polystyrene/polyethylene terephthalate (PET) thin film is appropriate for solar cell applications.

## Abstract 2

In the current research, the resist action of silver-doped polystyrene/polyethylene terephthalate (PET) solar thin film towards laser irradiation was observed.

## Graphical abstract

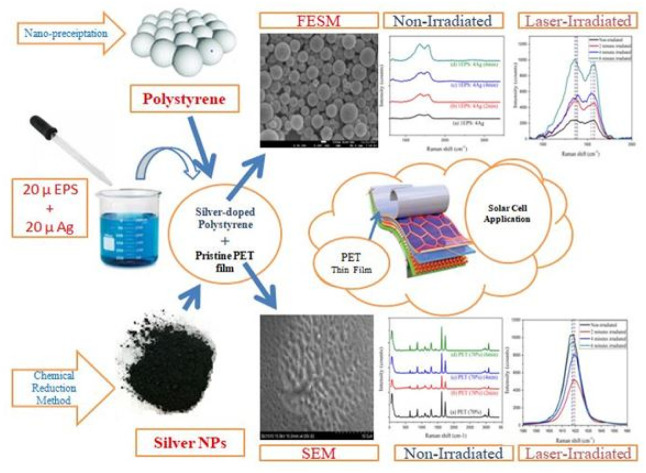

## 1 Introduction

Lithography is defined as the patterning, etching, and coating of many layers of thin films.^1^ The most widely used form of lithography is photolithography. Photolithography is widely applied in semiconductor, capacitor, and IC (Integrated circuits) chip manufacturing industries. By using optical radiation, the desired pattern can be transferred from masks to the semiconductor wafers through photoresist layers.^2^ Photoresists can be divided into two types which are positive resist and negative resist. In the case of positive photoresist, the long chain polymer has broken into smaller and more soluble fragments and become chain scissoring.^3^ Examples of positive tone resistance are PMMA (polymethyl methacrylate) and ZEP520.^4,5^ In case of negative resists, the exposed regions will undergo cross-linking reaction (polymerization) to form three-dimensional structures which make it insoluble in developer solution.^6^ An example of negative tone resistance is polystyrene. Polystyrene resists is more etch resistance than PMMA, however, it has low sensitivity which limits its application to small-scale nano-patterning.^7^ It is found that when increasing the time of exposure, some of the resists exhibit a switching behavior, from positive resists to negative resists or *vice versa*.^8^ The switching behavior is known as the zwitter characteristic. The resists that show the zwitter characteristic include PMMA, PS, nitrocellulose, triphenylene, and poly(di-*n*-hexylsilane).^9^

This modified polymer has wide applications in the medical, optoelectronic, and electronic fields. Polystyrene has been important nonmaterial's in the area of materials science in the current decade. Polystyrene due to its low bandgap, quantum confinement effect, higher surface–volume ratio, and some exceptional physical and chemical characteristics have been examined in detail to use in electrical, optoelectronic, and solar cell thin film applications.^10^ As compared to semiconductors, polystyrene semiconductors exhibit environmentally friendly, high mobility, chemical and thermal stability, inexpensive, compatible, and easy processing with semiconductors for thin film solar cell applications. Silver Doped Polystyrene/Polyethylene Terephthalate (PET) thin films has been deliberated for several causes including higher theoretical capacity, safety, environmentally friendly, radiation resistance, chemical durability, non-toxicity, and also some exceptional characteristics like the optical and electronic characteristics of solar cell.^11^

EPS (expanded polystyrene) is a prominent and environment-friendly material for packing and construction moreover, EPS is a lightweight stiff soft foam with the best and most efficient thermal lagging and efficient resistance. The proficient electronic, thermal, and mechanical properties are impervious to humidity therefore EPS materials are resistive to water vapors.^12,13^ The life cycle assessment (LCA) of usually used EPS is based on external thermal insulation systems (ETICS). This assessment is a worldwide established method for accessing the material's effects on the environment. In the present era, the concept of sustainable progress had the most central part in thinking about future interrelationships between the economy, society, and natural resources. In civilized societies, development and progress can be attained sustainably which will ensure equal access to future generations. EPS is a plastic material derived from crude oil used in a wide range of applications. EPS (expanded polystyrene) is a green material. In the production of EPS materials, any toxic raw products are not utilized. The process of making EPS involves expanding the polystyrene granules into a cellular shape using pentane a non-CFC expansion agent. Moreover, support for its green environment friendly can be observed in the life cycle of expanded polystyrene. Expanded polystyrenes are 100% eco-friendly. EPS does not pollute the water or air with gases or hydrosoluble elements. EPS makes up only a tiny part of 0.1% of solid waste materials.^14^ EPS is not friendly for the growth of bacteria and fungi. The transformation progression consumes very small energy and does not produce waste. The EPS user and producer do not bear any risk to the environment or health. EPS is lightweight foam with good impact resistance, absolute water, thermal insulation, load-bearing capacity at a light weight, vapor barrier, air tightness for controlled environments, extensive life, minimum cost maintenance, and economic and efficient construction. EPS does not react with salt or alkali solution as compared to other thin film materials such as nickel oxide thin film. EPS thin film has a better temperature coefficient, small grain size, and efficient conductive feature as compared to another thin films.^15^

Numerous techniques are applied to fabricate silver nanoparticles, such as sol–gel, pyrolysis, magnetron sputtering, hydrothermal, thermal oxidation, and co-precipitation. Among these synthesis methods, the chemical is a well-known fast and facile production method to fabricate silver-doped polystyrene nanostructures due to its well-defined size and shape, scalable fabrication, and easy manipulation. Though, the chemical method is very rarely applied for the synthesis of nanomaterials. The current literature explains the chemical production of silver-doped polystyrene for solar cell applications.^16^

An accurate description of the morphology and crystalline behavior with Nd:YAG pulsed laser of polystyrene and polyethylene terephthalate (PET) thin film is essential since it plays a proficient role in solar cells. As a result, the primary aim of the present work is to provide some important and significant additional information to the presented data on polystyrene and polyethylene terephthalate (PET) thin film. We have shown the results of investigations on the resisting behavior of PET and silver-doped polystyrene. The source that is used for exposure is Nd:YAG pulsed laser. Each sample was irradiated for 2 minutes, 4 minutes, and 6 minutes respectively. The findings reveal that there is a decreasing variation in the crystallinity of PET film after laser irradiation. Furthermore, the XRD, FESEM, SEM, and Raman spectroscopy results of polystyrene and polyethylene terephthalate (PET) thin to determine is superior for solar device applications.

## 2 Experimental details

**Fig. 1:**
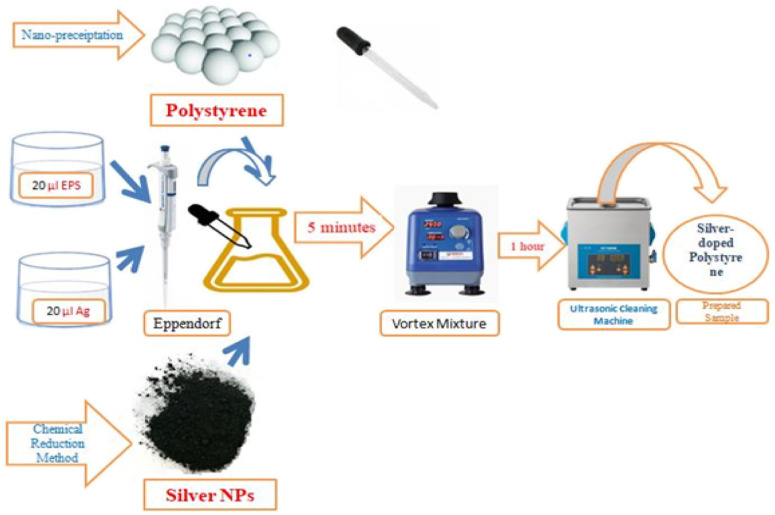
Experimental diagram of sample preparation of silver-doped polystyrene.

The polystyrene nanoparticles had been successfully synthesized *via* the nano-precipitation method as clarified in previous research^17^ while the silver nanoparticles were produced through the chemical reduction method by using sodium borohydride as the reducing agent.^18^ The silver-doped polystyrene (Ag-doped PS) was prepared in two ratios, 1 (EPS) : 1 (Ag) and 1 (EPS) : 4 (Ag). The sample was made by putting the 20 μl EPS (expanded polystyrenes) and 20 μl Ag (silver) into the Eppendorf by using a micropipette. Next, the sample was mixed by using Vortex Mixer and ultrasonic cleaning machine for 5 minutes and 1 hour respectively. Vortex Mixer is a device used to mix a small amount of liquid as shown in Fig. 1. The ultrasonic cleaning machine is used to make sure that the sample is well mixing. These steps were repeated to synthesis Ag-doped PS with ratio 1 (EPS) : 4 (Ag). These solutions were coating on a silicon substrate by using dropping method and left to evaporate in room temperature for one day.

Two commercial PET (Polystyrene/Polyethylene Terephthalate) window tint solar films with a degree of shades of 70% (light shade) and 35% (black-out shade) were purchased. Therefore, the samples were irradiated by using Nd:YAG (neodymium-doped yttrium aluminum garnet; Nd:Y_3_Al_5_O_12_) pulsed laser. The samples were irradiated with different energy densities which are manipulated by the time of exposure (2 min, 4 min, and 6 min). The laser irradiation parameters used in the experiments are presented in Table 1. The samples that have been irradiated were characterized by using several instruments. Field emission scanning electron microscope (FESEM), scanning electron microscope (SEM), Raman spectroscopy, and powder X-ray diffraction (XRD) were employed to investigate the surface morphology and structural properties before and after laser irradiation.

**Table 1:** Irradiation parameters for the laser exposure

Irradiation parameters	Value
Energy	700 mJ
Wavelength	1064 nm
Pulsed repetition rate (Hz)	2 Hz
Atmosphere	Air
Substrate temperature	Room

## 3 Results and discussion

### 3.1 Structural analysis

**Fig. 2:**
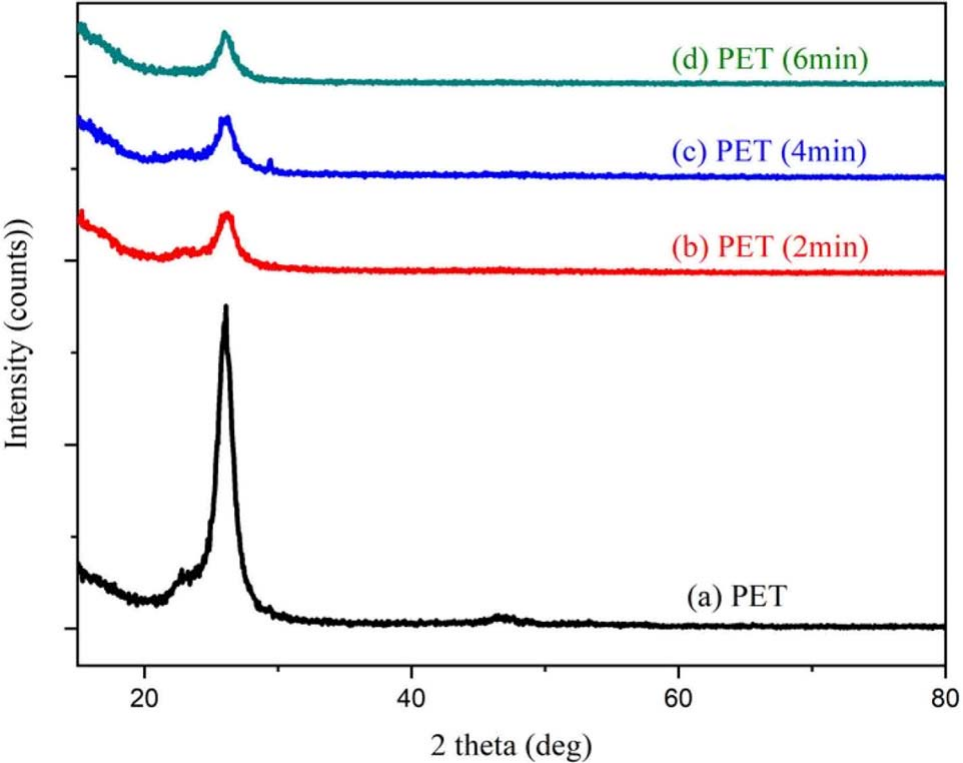
XRD spectrum of pristine PET and laser irradiated PET with a laser irradiation time of 2 min, (c) 4 min, and (d) 6 min.

The crystallinity of Ag-doped PS at different laser bombardment times has been studied by using XRD. Fig. 2 shows the XRD spectrum of pristine PET film with light shade (70%) and laser-modified PET film with different bombardment times. The semi-crystal form of PET film had not been changed. However, it is observed that the peak at around 2*θ* = 26° tends to decrease after being bombarded with Nd:YAG pulsed laser. This means that the crystallinity of PET film was decreased after laser irradiation.

The Debye–Scherrer formula can be used for the calculation of the average crystalline size from the peak.^19^1
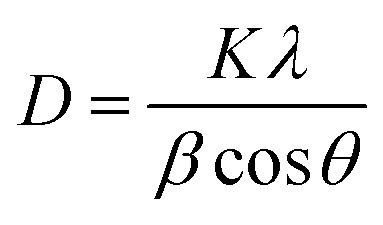
where ‘*K*’ is the shape factor which has a constant value of 0.94, ‘*λ*’ is the wavelength of falling X-ray radiation and ‘*β*’ is the full width at half the maximum value of the diffraction angle *θ*.

The X-ray density (*ρ*_XRD_) can be calculated by following relation.^20,21^2
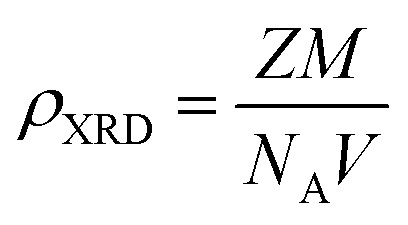
where ‘*Z*’ is some molecules per unit cell, ‘*M*’ is molecular weight, ‘*N*_A_’ is Avogadro's number and ‘*V*’ is unit cell volume, and by its value from eqn (2) can be put in eqn (3), then it will be obtained as:3
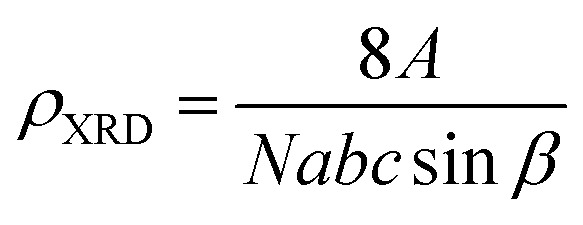
where *M* is the molecular weight of the particular oxide and *abc* sin *β* is the volume of the monoclinic unit cell.

### 3.2 Morphological analysis

#### 3.2.1 Silver-doped polystyrene

**Fig. 3:**
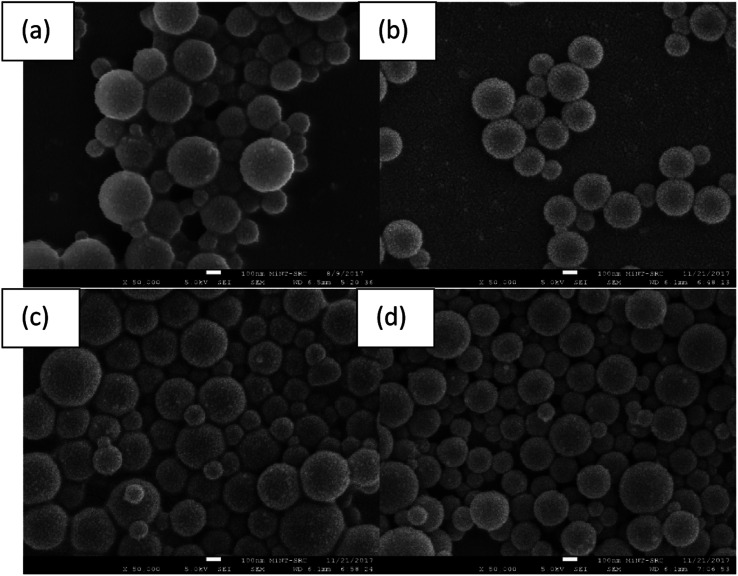
FESEM micrographs of (a) silver-doped polystyrene (1EPS:4Ag) and (b) with a laser irradiation time of 2 min, (c) 4 min, and (d) 6 min.

Fig. 3 shows the FESEM images of the non-irradiated and laser-irradiated silver-doped polystyrene at different times. The average particle size of silver-doped polystyrene irradiated at 0 minutes, 2 minutes, 4 minutes and 6 minutes are 320 nm, 242 nm, 336 nm, and 304 nm respectively. Successively size degradation and promotion after irradiation proved that the Ag-doped PS has been modified by laser exposure. Besides that, at 2 minutes of exposure, the bonding between the nanoparticles becomes less tightly which may be suggested to undergo chain-scission (positive resist). However, the nanoparticles tend to bind more closely at 4 minutes of laser exposure which may indicate that cross-linking and polymerizing process (negative resist). At 6 minutes of laser irradiation, the micrographs showed that there is a sign of a chain-scission process taking place because the nanoparticles dispersed much wider compared to the Ag-doped PS arrangement at 4 minutes of irradiation. Therefore, the Ag-doped polystyrene was suggested initially behaving as a positive resist and exhibiting successively negative and positive states.

#### 3.2.2 Polyethylene terephthalate (PET) thin film

**Fig. 4:**
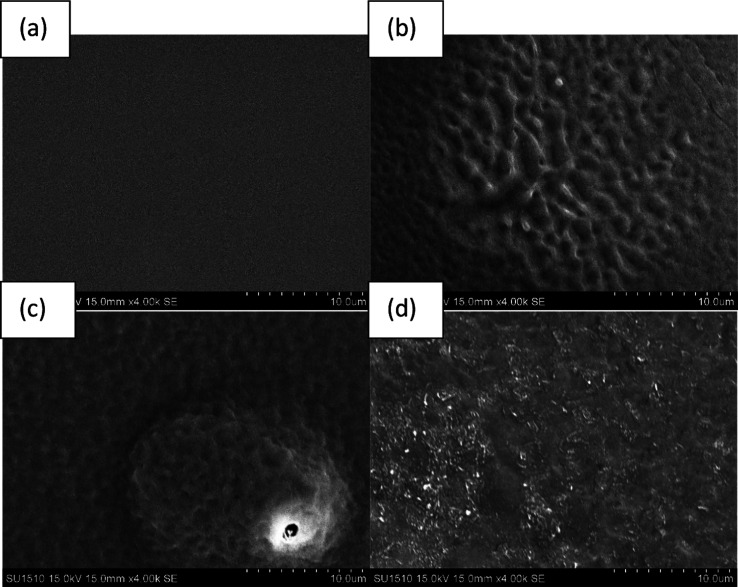
SEM micrograph of the (a) pristine PET film and (b) modified PET film with a laser irradiation time of 2 min, (c) 4 min, and (d) 6 min.

Fig. 4 shows the SEM micrograph of un-irradiated PET film and irradiated PET film at 2 minutes, 4 minutes, and 6 minutes of laser exposure respectively. The surface of the unirradiated PET film was flat and smooth as illustrated in Fig. 4(a). The surface roughness increased after 2 minutes of exposure and decreased when further bombarded the samples to 6 minutes which may be due to the change of chain bonding. These changes may be beneficial to the modification of wet ability and surface modification.

### 3.3 Raman analysis

#### 3.3.1 Silver-doped polystyrene

**Fig. 5:**
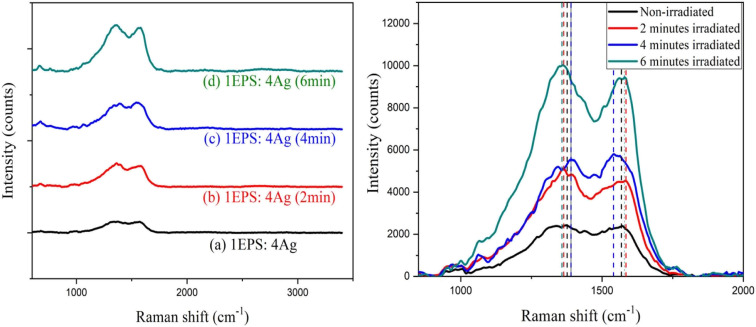
Raman spectra of (a) non-irradiated and irradiated silver-doped polystyrene (1EPS:4Ag); (b) bands at 1380 cm^−1^ and 1560 cm^−1^ in detail.

Raman spectroscopy, which is a very sensitive probe to the vibrations of the material and its local atomic arrangements, has been usually employed for the examination of the microstructural nature of the nano-materials. Raman scattering also gives important information about the bonds and structures of materials. More insight into the study of resist behavior was obtained by using Raman spectroscopy. Fig. 5(a) shows the Raman spectra corresponding to silver doped polystyrene (1EPS:4Ag) before and after irradiation at a different period while Fig. 5(b) shows the details of the bands at 1380 cm^−1^ and 1560 cm^−1^ upon irradiation. The changes of the bands at 1380 cm^−1^ and 1560 cm^−1^ upon irradiation were assigned to the forming of the carbonyl group. Two peaks were founded to shift closer at 4 minutes irradiation and become further apart at 6 minutes irradiation which may indicate the rearrangement of the polymer chains after irradiation. The band was broader and the intensity of scattered light increased as the laser irradiation time increased which may be due to the increasing carbonyl group forming and may be taken to represent a more crystalline state of the carbon resulting from an excessive laser dosage.

#### 3.3.2 Polyethylene terephthalate (PET) thin film

**Fig. 6:**
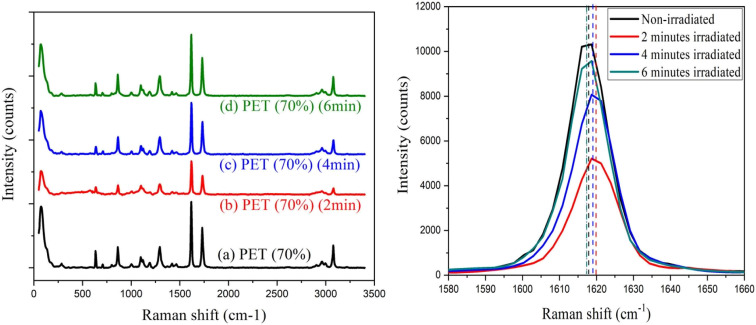
(a) Raman spectrum of pristine PET and laser irradiated PET with different bombardment periods; (b) Raman spectra of bands at 1615 cm^−1^ in detail.

Besides the morphological modification induced by laser irradiation, the chemical changes of the irradiated surface were inspected by Raman spectroscopy. Fig. 6(a) shows the Raman spectra for un-irradiated and irradiated PET film and Fig. 6(b) shows the details of the changes of bands at 1615 cm^−1^ (C–C ring stretching) upon laser bombardment. The Raman bands in the spectra of PET films were summarised in Table 2. It is observed that when the sample was irradiated for 2 minutes, this band shifted by around 2 cm^−1^ to a higher wave number and become narrower. Moreover, the intensity of scattered light detected become lower at 2 minutes of exposure. This may be due to the rearrangement of the polymer chains after irradiation. However, when the PET film was further irradiated for 6 minutes, the band tends to shift back to the lower wave number which is almost the same as the non-irradiated PET film. The bands become broadened and the intensity of scattered light detected become higher. These changes have shown the switching behavior of PET film to chain scission or cross-linking upon laser irradiation.

**Table 2:** PET Raman bands

Wavenumber (cm^−1^)	Types of vibration
1294	C(O)–O stretching
1615	Stretching C–C vibration
1730	Stretching C=O vibration
2960	Methylene groups adjacent to oxygen atoms
3077	Aromatic C–H bonds

## 4 Conclusion

The zwitter resists the action of silver-doped polystyrene and PET film was shown in this paper. When increasing the laser irradiation time from 2 minutes to 6 minutes, Ag-doped PS initially behaves as a positive resist and exhibits successively negative and positive states. Raman spectra showed the shifting of bands at 1380 cm^−1^ and 1560 cm^−1^ upon irradiation which may indicate the switching behavior of the polymer chains. The crystallinity was suggested to increase as the more carbonyl group formed upon irradiation. For PET film, the surface roughness was an increase after 2 minutes of laser exposure and decrease after 6 minutes of laser exposure. Besides that, Raman spectra revealed that the bands at 1615 cm^−1^ (C–C ring stretching) initially shift to a higher wave number and shift back to a lower wave number upon irradiation. This may be due to the cross-linking and chain-scissoring of the polymer chains. By knowing the amount of laser density required to induce the changes in polymer chains, the fabrication of a solar cell thin film by using a pulsed laser in one write is possible. The findings reveal that there is a decreasing variation in the crystallinity of PET film after laser irradiation moreover superior for solar device applications.

## Abbreviation

PET: Polystyrene/polyethylene terephthalate
IC: Integrated circuits chips
PMMA: Polymethyl methacrylate
Ag-doped PS: Silver doped polystyrene
EPS: Expanded polystyrenes
EPS: Expanded polystyrenes
FESEM: Field emission scanning electron microscope
SEM: Scanning electron microscope
XRD: X-ray diffraction

## Data availability statement

The data that support the findings of this study are available from the corresponding author upon reasonable request.

## Conflicts of interest

The authors declare that there is no conflict of interest.

## Supplementary Material
